# After morning, phew! A knowledge, attitudes, and practices survey related to emergency oral contraception in Thai pharmacists

**DOI:** 10.1186/s40545-023-00601-9

**Published:** 2023-08-01

**Authors:** Nattawut Leelakanok, Krittaphas Kangwanrattanakul, Arpa Petchsomrit, Bannawich Sapapsap, Tippawan Siritientong, Janthima Methaneethorn

**Affiliations:** 1 Division of Clinical Pharmacy, Faculty of Pharmaceutical Sciences, Burapha University, 169 Long-Hard Bangsaen Rd, Mueang, Chonburi 20131 Thailand; 2 Division of Social and Administrative Pharmacy, Faculty of Pharmaceutical Sciences, Burapha University, 169 Long-Hard Bangsaen Rd., Mueang, Chonburi 20131 Thailand; 3 Division of Pharmaceutical Technology, Faculty of Pharmaceutical Sciences, Burapha University, 169 Long-Hard Bangsaen Rd, Mueang, Chonburi 20131 Thailand; 4 Department of Food and Pharmaceutical Chemistry, Faculty of Pharmaceutical Sciences, Chulalongkorn University, Bangkok, Thailand; 5 Center of Excellence in Burn and Wound Care, Chulalongkorn University, Bangkok, Thailand; 6 Department of Pharmacy Practice, Faculty of Pharmaceutical Sciences, Naresuan University, Phitsanulok, Thailand

**Keywords:** Postcoital contraception, Health knowledge, attitudes, practice, Levonorgestrel, In-depth interview, Surveys and questionnaires

## Abstract

**Introduction:**

Pharmacists’ knowledge and attitude toward Emergency Oral Contraception (EOC) can affect users’ access to EOCs, especially where EOCs are provided by pharmacists without the need for prescriptions. We conducted a Knowledge, Attitudes, and Practice (KAP) survey on Thai pharmacists to better understand KAP related to EOCs and the correlation among KAP components.

**Methods:**

An in-depth interview, GoogleTrend search, and Pantip.com search were conducted. The findings, together with data from a previously published systematic review and meta-analysis, were used to develop KAP survey questions which were distributed online. Spearman’s rank correlation coefficient and linear mixed model were used to investigate the correlation and association among KAP components.

**Results:**

The in-depth interview with pharmacists showed that sex and unwanted pregnancy are very sensitive topics in Thailand. Sex and EOC education should be provided by parents and healthcare professionals at a young age. This agreed with opinions from Thai internet users that sex literacy was generally low and sex education was not provided adequately. From the total of 421 survey responses, Thai pharmacists had average knowledge, poor attitude, and average practice related to EOCs (median score = 51.02%, 21.81%, and 60.0%, respectively). The correlations between KAP in pharmacists were weak (ρ = 0.107–0.525, *p* < 0.02). Pharmacists who rated themselves as having higher scores in knowledge and attitude also rated themselves higher in the practice score. However, the total scores describing the knowledge of or attitude toward EOCs were not associated with EOC practice scores.

**Conclusions:**

In Thai pharmacists, self-rating KAP scores overestimated total KAP scores. The correlation among KAP components was weak. EOC knowledge and attitudes should be promoted, although this may not improve EOC practice in Thai pharmacists.

**Supplementary Information:**

The online version contains supplementary material available at 10.1186/s40545-023-00601-9.

## Introduction

Unintended pregnancies are a global burden in healthcare. The rate of unintended pregnancy, although in decline, is still high in developing countries [1, 2]. Emergency Oral Contraception (EOC) is one of the methods proposed for the prevention of unintended pregnancies [3, 4]. Unfortunately, women with unintended pregnancies usually do not use or are not aware of EOCs. Studies showed that inadequate knowledge of emergency contraceptives in users was associated with unplanned pregnancy [5–7]. Cultural beliefs, fear of side effects, lack of knowledge, and religion are also the reasons for refusing to use and provide EOCs [8].

The Knowledge, Attitudes, and Practices (KAP) theory states that knowledge and attitude are important factors in changing behaviors or practices [9]. Providing correct information on EOCs should lead to improving attitudes, thus improving behaviors that are related to EOC acceptance. However, a weak correlation between KAP has been criticized [10], so having correct knowledge of EOCs might not lead to positive attitudes or EOC use. In EOC users, this might not be the case, since literature shows that providing EOC knowledge to EOC users improves EOC use [11]. However, such information in healthcare professionals does not exist. Therefore, KAP in healthcare professionals is important to understand the barriers to EOCs from the provider’s end. Pharmacists increasingly involve with EOCs, since levonorgestrel EOCs are provided to patients by pharmacists without the need for prescriptions in several countries, e.g., Australia, Canada, China, Thailand, USA, and the UK [8]. Consequently, investigating KAP related to EOC in pharmacists is important for EOC promotion.

In Thailand, levonorgestrel EOCs are also available in pharmacies, although users can access the EOCs from hospitals or clinics. We assume that most of the EOCs are provided in pharmacies and users request EOCs directly from community pharmacists, albeit this has never been confirmed. Information on KAP related to EOCs in Thai pharmacists is not available [8], although Thai people may stereotypically perceive that KAP in Thai pharmacists is poor. For example, in the short film, Patcha is Sexy, a pharmacist refused to provide EOC for a teenager and a pharmacist replaces EOCs with a look-alike drug in the series, You Are My Makeup Artist. In this study, we conducted a cross-sectional online survey of Thai pharmacists to explore their baseline KAP. The correlation of KAP related to EOCs and factors that explained the KAP were also investigated.

## Methods

This research consisted of three major parts. The first one was to collect the necessary information for the KAP survey construction. We conducted in-depth interviews, Google Trend searches, and Pantip.com searches to collect the data in this phase. The second part was to design and evaluate KAP survey questions. To construct the KAP survey, we compared data from the first part with data from a previously published systematic review and meta-analysis [8]. These questions were then evaluated by experts. In the last phase, KAP cross-sectional survey was distributed to collect data on KAP related to EOCs and the correlation among KAP components.

### Collecting data for survey construction: in-depth interview

The protocol for the research was approved by Burapha University Institutional Review Board (IRB1-003/2565). This in-depth interview complied with the Consolidated Criteria for Reporting Qualitative Research. The first author is a registered pharmacist with an assistant professorship and had published a few qualitative studies in peer review journals. He trained 3 year five female pharmacy students to be Research Assistants (RAs). No assumptions were assumed, and the RAs were not personally interested in the topic. Phenomenology was used in this study. The first author and his RAs designed semi-structured interview questions for pharmacists. The questions were also reviewed by two independent registered pharmacists. The interview questions were pilot-tested on five pharmacy students and modified accordingly (Additional file 1: Data 1).

The interview occurred from October 2021 to March 2022. The participants were selected by convenience sampling and approached face-to-face. The target number of enrollments was approximated at 25 [12] or when the theme was saturated. Currently registered pharmacists with active pharmacist licenses were included. The interview was scheduled later online via GoogleMeet (Google LLC, USA) because of the coronavirus disease pandemic. Participants were free to select their interview place. No relationship between researchers and interviewees was established before the interview. However, participants knew that the interview was required for the graduation of the RAs. The RAs conducted and audio-recorded all interviews. Field notes were taken during the interview.

The RAs created transcripts from the interviews. The first author and the RAs read and discussed the transcript together. Comments on the transcript were generated in Microsoft Word 365 (Microsoft Corporation, USA). The comments were categorized and collected as themes by consensus. Themes were used to further improve the interview questions until no new themes emerged. The transcript was not returned to the interviewees, so no feedback was obtained. The themes and illustrative quotes were translated into English by the first author.

### Collecting data for survey construction: Google Trend searches

Words related to EOCs were searched in Google Trend (Google LLC, USA) from 2004 to February 2022. Search terms in Thai including emergency oral contraceptive, and morning-after pill were searched by limiting the location in Thailand, while search terms in English (emergency contraception (pill), morning-after pill, levonorgestrel, Postinor, and ulipristal) were searched worldwide. Related queries retrieved from each search were compiled in Microsoft Excel 365 (Microsoft Corporation, USA). The list of queries was generated after redundancy was removed.

### Collecting data for survey construction: Pantip.com searches

https://pantip.com/ is the largest online Thai community since 1997. The website, although it is not a search engine, has a function that allows searching for related topics on the webpage and its forum categories that are discussed. However, the maximum number of retrieved forums is 20,000 per search. We used a Thai search term for morning-after pills to search from inception to April 1st, 2022. Questions and concerns related to EOCs were manually collected, transferred to Microsoft Excel 365, and categorized.

### Survey question design and evaluation

The protocol for the research was approved by Burapha University Institutional Review Board (IRB1-085/2565). Data from the in-depth interview, Google Trend searches, Pantip.com searches, and a previously published systematic review and meta-analysis [8] were used for the design of the KAP survey. We aimed to collect data on the demographics, and EOC-related KAP of pharmacist participants. Knowledge questions were closed-ended with multiple-answer choices. Self-rating KAP; self-rating general attitudes (e.g., religiousness, openness to sex discussion); attitudes; and practice questions were answered as five-point Likert scales. The questions were reviewed and modified by all authors. To assess content validity, the survey questions were rated by five, a recommended number from the literature [9], non-author pharmacist experts using a five-point Likert scale, where 5 = highly relevant, 4 = relevant, 3 = neutral, 2 = mostly not relevant, and 1 = totally not relevant. Then, the index of Item Objective Congruence (IOC) was calculated for each question. In addition, the expert panels also checked and commented on the appropriateness of survey questions and their response options. After the modification according to the expert comments, the third author transformed the survey into Google Forms (Google LLC, USA.), which could be accessed only by the first and third authors. Google Forms was set to not include multiple submissions from the same individual. The survey was sent to 10 pharmacy students for their opinion on the comprehensibility of the questions and the feasibility of the online survey.

### Data collection via KAP cross-sectional survey

The number of registered pharmacists reported by the Thai Pharmacy Council on December 1st, 2021, was 45,707. We use Open Epi online version 3.01 to calculate the sample size using its default setting and alpha of 0.05. With a 10%-excess number of participants, we aimed to collect data from 420 pharmacists. We included all pharmacists of any age with an active pharmacist license who were willing to answer the survey. The advertising material was posted in online communities of Thai pharmacists, e.g., a Facebook page Pharmacafe with approximately 26,800 members, and groups of pharmacists in the Line application, to ensure that we reached the target samples. We recommended participants answer the survey questions online on their computers to reduce fatigue. This survey was reported according to A Consensus-Based Checklist for Reporting of Survey Studies.

### Data and statistical analysis

Data were exported and cleaned in an Excel worksheet. Missing data and non-response errors were assumed to be missing completely at random and were handled by exclusion. Imputation and adjustment for the non-representativeness of the sample were not conducted. We defined total scores as a sum of correct answers from selected questions regarding knowledge, attitude, or practice, while self-rating scores were 5-scale Likert scores from where the participants rated themselves on their knowledge, attitude, or practice. Attitude and practice questions that were used for calculating the total attitude and total practice scores were discussed and reached by consensus among authors.

Descriptive statistics were performed using Microsoft Excel 365. Inferential statistics were conducted using SPSS (IBM SPSS Statistics 28.0.0.0, IBM, USA). We planned a priori to see the correlation among KAP by calculating Spearman’s rank correlation coefficient. The coefficient of less than 0.4 and 0.7 were classified as weak and medium correlations, respectively [13]. Associations between EOC dispensing practice and 17 independent variables including demographics (age, degree, marital status, opinion on unintentional pregnancy, practicing duration, type of pharmacy practice, religions, self-rating judgment, self-rating religiousness, self-rating openness to sex discussion, and sex); a total score and self-rating KAP, were analyzed by the linear mixed model. The number of Subjects per Variable (SPV) in this study was approximately 24 (420/17) which was higher than 10, the recommended lowest SPV [14]. No adjustment for the non-representativeness of the sample and sensitivity analysis was conducted.

## Results

Overall, 64, 26, 75, and 115 questions emerged from the in-depth interview, Google Trend searches, Pantip.com search, and the previously published systematic review and meta-analysis [8], respectively. After eliminating repeated questions, we obtained 57 knowledge-related questions and 103 attitude-related questions. The questions were evaluated by the experts with an IOC of > 0.7. The survey was sent to registered Thai pharmacists. Four-hundred and twenty-seven responses were obtained and used for descriptive and linear-regression statistical analysis.

### Collecting data for survey construction: in-depth interview

We included 17 pharmacists in the in-depth interview. Although we did not reach the planned sample size because of the heavy lockdown from the coronavirus disease, we reached theme saturation. All the pharmacists that we reached out to agree to participate in the interview. All interviews were conducted online by all three RAs without the presence of other people. The median interview duration was 15 min (interquartile range 5–15 min). No repeated interview was conducted. The median age of interviewees was 35 years, and the median practicing duration was 3 years. Most of the interviewees were female, Bachelor’s degree graduates, community pharmacists, and Buddhists (Table 1).

**Table 1:** Demographics of pharmacists who participated in the qualitative part of this study

	Age	Sex	Profession	Highest degree^a^	Working duration (Years)	Religious
1	25	Female	Community and hospital	Bachelor’s	1	Buddhism
2	25	Female	Community	Bachelor’s	1	Islam
3	25	Female	Community	Bachelor’s	1	Buddhism
4	26	Female	Medical representative	Bachelor’s	2	Buddhism
5	26	Male	Hospital	Bachelor’s	1.75	Buddhism
6	27	Male	Community	Bachelor’s	3	Buddhism
7	26	Female	Medical representative	Bachelor’s	1.25	Buddhism
8	35	Female	Community	Bachelor’s	10	Buddhism
9	48	Female	Community and education	Master’s	23	Buddhism
10	53	Female	Community	Master’s	28	Buddhism
11	26	Male	Community and education	Bachelor’s	2	Buddhism
12	36	Female	Community and education	Doctor’s	13	Buddhism
13	37	Male	Community and hospital	Master’s	5	Buddhism
14	37	Female	Regulation	Doctor’s	3	Buddhism
15	38	Female	Hospital	Master’s	15	Buddhism
16	38	Male	Community and education	Doctor’s	8	Buddhism
17	40	Female	Community	Master’s	6	Buddhism

^a^Bachelor’s degrees include a Bachelor of Pharmacy and a Doctor of Pharmacy. A doctor’s degree refers to a Doctor of Philosophy

All themes are illustrated in Table 2. Briefly, Thai pharmacists agreed that sex and unwanted pregnancy is still a very sensitive topic among patients and pharmacists. In addition, pharmacists agreed that sex and EOC education should be provided at a young age and families should be involved in providing the education. Some pharmacists perceived that using EOCs was an act of responsibility, while some still had negative attitudes toward EOC use. Some pharmacists expressed concern had their children used EOCs. Interestingly, inaccurate information on contraception and EOCs was found during the interview.

**Table 2:** Theme emerged during the semi-structured in-depth interview

Category	Theme	Illustrative quote
Knowledge and attitudes toward sex	Sex talk is still related to shyness	(P8) People, mostly teenagers, are shy about sex. This is because of communication. We fail to let them know that having sex is normal. Sexually transmitted infection is also another reason for making people shy to talk about it
	Sex should become normalized	(P9) We cannot prohibit people from having sex. We have an environment that promotes people to have sex more than we ever had (P10) Parents should accept that sex is normal in teenagers. Their kids have changed. The world has changed. Parents should invest more in sex education. Make them understand. Do not scold them
Knowledge and attitudes toward contraception	There are false beliefs among pharmacists about contraception	(P8) Most of my patients do not know how to monitor their menstrual cycle, so they do not know or are not confident in using the fertility-awareness method. When I tell my patient that they do not need the EOCs, since they are in the “safe period”, they do not believe me. They risk taking EOCs instead of using the fertility-awareness method
	Unintentional pregnancy is taboo	(P10) Males should also know about contraception, but they do not care. They should not let their girls frequently have morning-after pills, since the pills are bad. If the girls are pregnant, that is the males’ fault. Becoming pregnant not at the proper time can lead you to get expelled from school or work. This leads to excessive guilt and depression like they committed hideous sin. Pregnant women with undesired pregnancies may commit suicide or abortion
Knowledge and attitude toward emergency oral contraceptives (EOCs)	There are false beliefs among pharmacists about EOCs	(P1) High hormone levels in the morning after pills can cause severe side effects, such as ovary cancer, or uterine cancer. This should be announced to the public (P3) Levonorgestrel should not be used in overweight and obese people, since it lacks efficacy (P4) If pharmacists gave EOCs to their patients without thinking, it could put their patients at risk for cancer (P5) I do not like people who use EOCs frequently. It is stupid. It is unresponsible to themselves and their unborn child, since the efficacy of EOCs is reduced after two doses (P9) Using EOCs every day decreases their efficacy. Are you ok if the efficacy of ECOs is tenfold lower than combined oral contraceptives (COCs)?
	Knowledge of EOCs should be promoted	(P1) People do not have education about sex like how we have, so it is our job to educate them. Google is full of knowledge and garbage. I think EOCs should be taught in schools (P2) Sex education should be compulsory and provided to all Thai people. The content does not have to be in-depth but covers all the necessities. The more the people know, the fewer the problems (P6) I agree with having sex education since menarche (P7) Sex education should be provided in high school. However, it is not provided in a lot of schools. It would be nice if the family teaches their kids about sex
	Knowledge of the EOC mechanism of action affects whether pharmacists perceive EOCs as sinful	(P6) Using EOCs and sin? No, they are not related, even remotely. EOCs prevent fertilization and do not destroy lives. It is not the same as abortion (P8) EOCs cause unsuitable conditions for the eggs. I do not think it is sinful. It is sinful in some religions, maybe because the religions want to increase the human population
	Attitudes toward EOCs are negative	(P1) Only promiscuous people should have EOCs with them. Monogamous people do not need to (P2) I would not allow my kid to use EOCs. It should not be used according to religion. However, I may have allowed it if she was raped or not ready (P13) EOCs are not as dangerous as we use to think (P14) In Thailand, attitudes toward EOCs are quite negative. Users are shy. Some pharmacists are not professionals. There is also a lot of misunderstanding (P16) Those who use EOCs are promiscuous. In case guys buy EOCs, their female partner might not be fully willing to have sex
	EOCs are related to protection during sex	(P2) EOCs may lead to unprotected sex (P10) Using EOCs is already the protection (against pregnancy)
Factors affecting EOC use	Access	(P9) There are a lot of drug stores in the cities, so access is very easy. This is not the case in rural areas
	Parents	(P3) If my kids used EOCs, I might have not been that open-minded. I would feel bad. I would think why did they not use other methods? (P8) What is the cause for not knowing EOCs? Parents. They should also teach their children. They should not see sex as a bad habit. They should not scold their kids but educate them (P9) Parents do not want their children to use EOCs. I think parents are disappointed about their kids having sex more than about their kids using EOCs. The parents are afraid of unwanted pregnancy. Thai people are not that open-minded about this issue. Teenagers should take a long time to see each other before having sex
	Partner	(P6) The male partner has a lot of impact on whether a girl would use EOCs. The male partner may not want to use a condom or prefer to have unprotected sex. The female partner may not have a say in the way they have sex (P10) The male partner does not want to use a condom. The female partner does not want to use COCs. Both are lazy (P16) Some guys think that they are couples. Why do they need to use condoms? Using condoms means untrust or despisal for them. Some girls who are blindly in love agree not to use condoms and use EOCs instead
	Religion	(P2) Islam prohibits sex in people who are not married, even if they are in a serious relationship. Therefore, EOCs can be used only after medical consideration (P3) Education makes people believe that EOCs should not be used. People think that sex is bad. The elderly say that using EOCs is sinful but I think that it is normal (P4) Using EOCs does not concern religions. We are far from that point. Well, maybe except for a few religions though
	Social norm	(P4) Thai culture does not open to sex (P9) The norm is very strict about sex. I once wanted to conduct a research project (about EOC use in teenagers), but it was not allowed by the ethics committee. We are not that open. I would be so glad if a high schooler come and buy EOCs from me. We cannot force them to abstain from sex. Sex education is a better option
Responsibility	Using EOCs shows responsibility	(P1) Parents should not scold their kids when they use EOCs, since nothing is wrong. It is good that they are responsible for what they have done. I am ok with high schoolers who buy EOCs. They are responsible for themselves (P3) Do not want to be pregnant and do something about is already responsible (P7) Taking EOCs is protecting oneself. It is better than letting a child be born without love and support (P12) Using EOCs shows self-care but it would be better if condoms or COCs are used, since condoms and COCs show that the sex is planned
	Pharmacists are responsible for providing EOC knowledge	(P1) It is our job to let them know that EOCs cannot be used for abortion (P4) EOCs should be sold by pharmacists. Sellers should know and give buyers the knowledge (P11) If pharmacists take the patient history and think that they should not use EOCs, that is ok. But I have a very negative opinion of those who refuse to dispense EOCs for personal reasons. We have the responsibility. We are educated and obligated to use our knowledge with our patients
	Refusing EOC dispensing is not acceptable	(P12) I think that pharmacists who refuse to dispense EOCs must be very old and conservative. Such a pity

*EOCs* emergency oral contraceptives, *COCs* combined oral contraceptives

### Collecting data for survey construction: Google Trend searches

We found 665 queries about levonorgestrel and ulipristal acetate EOCs in EOC users. After removing redundant queries, 221 EOC-related queries were categorized into 34 topics and used in 26 survey questions (Tables 3, 4, 5).

**Table 3:** Survey questions for EOC knowledge and their sources, answers, references for the answers, and scores

Survey question	Source of question	Answer	Possible score	Number of correct answers^b^ (*n*/total)
What are emergency oral contraceptives (EOCs)^a^ that are commercially available in Thailand?	–/–/P/S	Levonorgestrel	0–1	337/421
What are the tradenames of the EOCs that are commercially available in Thailand?	G/–/–/S	Postinor^®^, Madonna^®^, Maple Forte^®^	0–3	155/421
What are the indications of the EOCs?	–/I/P/S	We decided not to include the scores of this question, because all answer choices are defined as unplanned unprotected sex	N/A	–/421
What is the dosage form of the EOCs?	–/–/–/S	Film-coated tablets	0–1	291/421
What is the EOCs’ mechanism of action?	G/I/P/S	Preventing the ovulation EOCs act by inhibiting or postponing ovulation (Gemzell-Danielsson et al., 2013). Levonorgestrel must be taken within a window period after the selection of follicle but before the luteinizing hormone surge (Gemzell-Danielsson et al., 2013). In addition, the conception rate of taking EOCs after ovulation does not differ from the placebo (Endler et al., 2022). This indicates the insignificant of other proposed mechanisms of actions of EOCs. Other studies also defy other mechanisms of action of levonorgestrel. For example, a study shows that gene expression in the endometrium does not change after taking levonorgestrel EOCs (Vargas et al., 2012). Another study shows that taking levonorgestrel EOCs after ovulation does not prevent implantation (Endler et al., 2022)	0–1	22/421
How do you access the information on EOCs?	–/–/P/S	There is no correct answer to this question. More than 50% of pharmacist access EOC information from package inserts and practice guidelines	0	–/419
What is the strength of the EOCs?	G/I/P/S	0.75 mg, 1.5 mg	0–2	229/421
What is the total dose of the EOCs for one coitus?	G/I/P/S	1.5 mg	0–1	359/421
EOCs can be taken two tablets every …… hour apart	G/I/P/S	12 h (2 points) or 24 h (1 point) Levonorgestrel EOCs are approved as two doses of 0.75 mg, 12 h apart, or a single dose of 1.5 mg. However, Tmax, Cmax, and AUC derived from taking levonorgestrel 12 h and 24 h apart are not significantly different (Johansson et al., 2002). In addition, a randomized controlled trial in 2071 women showed that taking 0.75 mg of levonorgestrel 120 h after coitus, either 12 h or 24 h apart, is effective (Wai Ngai et al., 2005)	0–2	375/421
The dose of EOCs must be adjusted when the users are heavier than …… kg	G/I/P/S	There is not enough evidence for the answer Although there is a recommendation to double the dose of EOCs in patients who weigh more than 70 kg or have a BMI of more than 26 kg/cm^2^ or 30 kg/cm^2^, the recommendation is questionable (Gemzell-Danielsson et al., 2015; Kardos, 2020)	0–1	220/421
If the dose of EOCs must be adjusted when the users are heavier, the dose should be adjusted to ……. mg/coitus	G/I/P/S	There is not enough evidence for the answer	0–1	214/421
The EOCs should not be used for more than how many boxes (dose/coitus) per month?	G/I/P/S	There is not enough evidence for the answer	0–1	66/421
What is the maximum number of EOCs in boxes that can be taken continuously?	G/I/P/S	There is not enough evidence for the answer	0–1	132/421
What is the maximum number of EOCs in boxes that can be taken in a lifetime?	–/–/P/–	There is not enough evidence for the answer	0–1	263/421
Is a pregnancy test required before using EOCs?	–/–/P/S	No	0–1	268/421
EOCs are used before or after coitus?	G/–/P/S	After only EOCs are approved for postcoital use. In addition, there are no studies investigating the effectiveness of 1.5 mg of levonorgestrel before intercourse (Halpern et al., 2014; Raymond et al., 2011)	0–1	312/421
The first dose of EOCs should be taken within how many hours after coitus?	G/I/P/S	72	0–1	210/421
The first dose of EOCs should be taken, as late as possible, within how many hours after coitus?	G/I/P/S	120 (Wai Ngai et al., 2005)	0–1	350/421
The earlier the first dose of EOCs, the better the contraception efficacy. Is this true?	G/I/P/S	True (Matyanga & Dzingirai, 2018)	0–1	406/421
What is the restriction period for another coitus, in hours, after the use of EOCs?	G/–/P/–	There is no restriction	0–1	223/421
What is the efficacy of 0.75 mg EOCs/coitus?	–/I/P/S	The Pearl Index of this method is reported as 22.4 (Taylor et al., 2014). Therefore, an efficacy rate of approximately 78% can be expected	0	The answer was^d^ 52 ± 23% (-/413)
What is the efficacy of 1.5 mg EOCs/coitus?	–/I/P/S	The Pearl Index of this method is reported as 6.8 (United Nations Development Programme et al., 2000). Therefore, an efficacy rate of approximately 93% can be expected	0	The answer was^d^ 81 ± 11% (–/414)
What is the efficacy of combined oral contraceptive pills?	–/I/P/S	The Pearl Index of this method is reported as 0.29–2.86 (Trussell & Portman, 2013). Therefore, an efficacy rate of approximately 97% can be expected	0	The answer was^d^ 92 ± 10% (–/416)
What is the efficacy of condoms?	–/I/P/S	The Pearl Index of this method is reported as 1.26 (Zhao et al., 2014). Therefore, an efficacy rate of approximately 99% can be expected	0	The answer was^d^ 92 ± 12% (–/415)
What is the efficacy of the lactational amenorrhea method?	–/I/P/S	The pregnancy rate of this method is reported as 0.5–1.5 (Trussell, 2009). Therefore, an efficacy rate of approximately 98% can be expected	0	The answer was^d^ 73 ± 26% (–/416)
Are EOCs effective after ovulation?	G/I/P/S	No (Noé et al., 2011)	0–1	65/421
If vomiting occurs after taking EOCs, another tablet must be taken when taking EOCs and vomiting are how many hours apart?	–/–/P/–	2 h The time to maximum concentration of levonorgestrel is approximately 2 h (Johansson et al., 2002; Kives et al., 2005; Kook et al., 2002)	0–1	196/421
The more frequently the EOCs are taken, the less the EOCs are efficient. Is this true?	–/I/P/–	No The probability of contraception failure over time is well-discussed by Trussel in 2009 (Trussell, 2009)	0–1	136/421
EOCs can be active for how many hours after the dose is complete?	G/–/P/S	72 h We cannot speculate on the effective duration of levonorgestrel after its oral absorption from its precoital study, since there are no studies investigating the effectiveness of 1.5 mg levonorgestrel before intercourse (Halpern et al., 2014; Raymond et al., 2011). However, an in vitro study shows that levonorgestrel can inhibit sperm migration in cervical mucous for 12 h after the dose (Kovacs et al., 2000). In addition, a pharmacokinetics study suggests that after completing the doses of levonorgestrel, contraception can be achieved for at least 3 days (Tremblay et al., 2001)	0–1	124/421
After the use of EOCs, if side effects occur, they usually occur for how many hours?	G/–/–/–	There is not enough evidence for the answer A pharmacokinetics study suggests that after completing the doses of levonorgestrel, plasma levonorgestrel can remain above its effective level for longer than 4.5 days (Tremblay et al., 2001). However, there is no study supporting the window period for levonorgestrel side effects after its last dose	0–1	30/421
What is the relationship between nausea and EOCs?	–/I/–/S	A side effect that does not require cessation of EOCs Nausea is one of the common but not serious adverse events reported during the use of levonorgestrel EOCs (Leelakanok & Methaneethorn, 2020)	0–1	401/421
What is the relationship between hypertension and EOCs?	–/–/–/–^c^	A side effect that requires cessation of levonorgestrel (American Pharmacist Association, 2018)	0–1	86/421
What is the relationship between ectopic pregnancy and EOCs?	–/I/P/S	Not enough evidence to support such a relationship/unrelated The evidence on the association between levonorgestrel and ectopic pregnancy is still conflicting (Leelakanok & Methaneethorn, 2020). WHO states that people with a history of ectopic pregnancy can still use progestin-only contraception or a levonorgestrel intrauterine device (Altshuler et al., 2015)	0–1	45/421
What is the relationship between breast tenderness and lactation and EOCs?	–/–/P/S	A side effect that does not require cessation of EOCs Breast tenderness and lactation are common but not serious adverse events reported during the use of levonorgestrel EOCs (Leelakanok & Methaneethorn, 2020)	0–1	257/421
What is the relationship between diabetes mellitus and EOCs?	–/–/–/–	A side effect that requires cessation of levonorgestrel (American Pharmacist Association, 2018)	0–1	20/421
What is the relationship between breast cancer and EOCs?	–/I/P/S	Contraindication Progestin-only contraception or levonorgestrel intrauterine device is contraindicated in people with breast cancer (Altshuler et al., 2015). It is prudent to assume that people with breast cancer should also not use levonorgestrel EOCs	0–1	265/421
What is the relationship between infertility and EOCs?	–/I/P/S	Not enough evidence to support such a relationship/unrelated Levonorgestrel as an EOC does not affect the menstrual cycle or endometrial receptivity to implantation. In addition, the evidence does not support that levonorgestrel causes miscarriage or teratogenicity (Endler et al., 2022)	0–1	208/421
What is the relationship between severe hepatic diseases and EOCs?	–/–/–/–	Contraindication (American Pharmacist Association, 2018; Vrettakos & Bajaj, 2022)	0–1	32/421
What is the relationship between thromboembolism and EOCs?	–/–/–/S	A side effect that requires cessation of EOCs Thromboembolism is one of the serious adverse events reported during the use of levonorgestrel EOCs (Leelakanok & Methaneethorn, 2020). WHO also recommended that the risk for thromboembolism in progestin-only contraception or levonorgestrel intrauterine device users outweigh the benefits of contraception (Altshuler et al., 2015)	0–1	50/421
What is the relationship between acne and EOCs?	–/–/P/S	A side effect that does not require cessation of EOCs Acne is one of the common but not serious adverse events reported during the use of levonorgestrel EOCs (Leelakanok & Methaneethorn, 2020)	0–1	283/421
Vaginal bleeding can occur within how many days after the use of EOCs?	–/–/P/–	1–7 days Two studies found that their participants had vaginal spotting 2 to 3 days after levonorgestrel usage (Hapangama et al., 2001; Okewole et al., 2007)	0–1	240/421
Vaginal bleeding can occur for how many days after the use of EOCs?	–/–/P/–	1–7 days One study reported that intermenstrual bleeding lasted for an average of 2.4 days (range 1–7) (Gainer et al., 2006)	0–1	249/421
What causes the bleeding after the use of EOCs?	–/–/P/–	Bleeding after EOC use is a side effect (Gainer et al., 2006)	0–1	221/421
What are the differences between vaginal bleeding after the use of EOCs and menstruation?	–/–/P/–	Vaginal bleeding is lesser in amount, thickness, and darkness (Gainer et al., 2006; Okewole et al., 2007)	0–3	17/421
After the use of EOCs, the menstruation cycle can be early or delayed for how many days?	G/I/P/S	Two days early to 2 days late, in general In one study, more than 50% of the participants had menses within 2 days after taking levonorgestrel (von Hertzen et al., 2002)	0–1	45/421
Using EOCs during pregnancy can cause teratogenicity. Is this true?	G/I/P/S	No	0–1	124/421
Using EOCs during pregnancy can cause abortion. Is this true?	G/I/–/S	No	0–1	178/421
What is the relationship between levonorgestrel and CYP450?	–/–/P/S	Levonorgestrel is a CYP3A4 substrate (Sunaga et al., 2021)	0–1	133/421
Patients using EOCs can be immunized with the COVID-19 vaccine. Is it true?	G/–/P/–	Yes Venous thrombosis was reported as an adverse event in users of RNA‐based, non‐replicating viral vector, and protein subunit COVID-19 vaccine (Graña et al., 2022). In addition, thromboembolism can be a serious side effect of levonorgestrel. However, the risk of thromboembolism in levonorgestrel users is the lowest (Leelakanok & Methaneethorn, 2020). Besides, levonorgestrel poses a lower risk of thromboembolism than COCs (Rott, 2019). Together with the fact that pregnancy poses higher risks for thromboembolism than using COCs and the risk for thromboembolism in COVID-19 patients is higher than people receiving COVID-19 vaccines (Lee et al., 2022), people using COCs are recommended to receive COVID-19 vaccine (Lee et al., 2022). Therefore, it is prudent to assume that EOCs can be immunized with the COVID-19 vaccine	0–1	373/421
Patients using EOCs can be immunized with the Human Papilloma Virus vaccine. Is it true?	–/–/–/–	Yes The vaccine is not known to cause serious adverse events. This vaccine should not be used in pregnant women. The only contraindication of the vaccine is a history of allergy to vaccine components (World Health Organization, 2017). Therefore, it is prudent to assume that EOCs can be immunized with the Human Papilloma Virus vaccine	0–1	303/421
Patients using EOCs can be immunized with the Hepatitis B virus vaccine. Is it true?	–/–/–/–	Yes The vaccine is not known to cause serious adverse events. The only contraindication of the vaccine is a history of allergy to vaccine components (World Health Organization, 2019). Therefore, it is prudent to assume that EOCs can be immunized with the Hepatitis B virus vaccine	0–1	299/421
Can combined oral contraceptive pills be used as EOCs?	–/–/P/S	Yes COC pills can be used as EOCs as in the Yuzpe regimen. However, this question is not included in scoring, since the question was not clear enough to make the participants understand whether COC can be used regularly (i.e., taking a tablet of COC) as EOC or COC must be used specifically (as in the Yuzpe regimen)	N/A	–/421
Have you ever heard of EOC abuse?	–/–/P/–	There is no correct answer to this question. Pharmacists have heard that EOCs are used for abortion, acne treatment, alopecia, delaying menstruation, hormone replacement in transgender, melasma, menstrual stimulation, and weight reduction	N/A	–/420

References for the answers are listed in Additional file 2: Data 2

*G* Google Trend, *I* interviews from the qualitative part, *P* Pantip.com search, *S* the systematic review and meta-analysis, *COC* combined oral contraceptive, *CYP450* cytochrome P450, *EOCs* emergency oral contraceptive

^a^Since the only EOC that is commercially available in Thailand is levonorgestrel, EOCs in all questions are intuitively referred to as levonorgestrel

^b^Only the participants with full scores are shown. If the question has multiple possible answers, participants who answer completely correctly without any incorrect answers are shown

^c^–/–/–/– means that the question is added by either the authors or the experts

^d^Presented as mean ± standard deviation

**Table 4:** Survey questions for EOC attitude and their sources, the frequency of the attitudes, and possible scores

Do you agree with the following sentences?	Source of question	Frequency (*n*)					Possible attitude scores
		Totally disagree	Disagree	Neutral	Agree	Totally agree	
People using condoms should also use EOCs	–/–/P/S	134^a^	127	59	66	31	0
Sterile people should also use EOCs	–/–/P/S	237^a^	107	46	23	6	0
People who have sex during menstruation should also use EOCs	–/–/P/S	130	134^a^	53	73	27	0
People who use combined oral contraceptives should also use EOCs	–/–/P/S	222^a^	126	30	31	9	0
People who ejaculate externally should also use EOCs	–/–/P/S	24	27	50	201^a^	117	0
People who use lactating amenorrhea method should also use EOCs	–/–/P/S	72	116^a^	101	111	19	0
It is dangerous for people using condoms to use EOCs	–/–/P/S	261^a^	99	37	20	1	0
It is dangerous for people who are sterile to use EOCs	–/–/P/S	226^a^	104	46	35	6	0
It is dangerous for those who have sex during menstruation to use EOCs	–/–/P/S	178^a^	122	66	41	10	0
It is dangerous for people who use combined oral contraceptives to use EOCs	–/–/P/S	66	50	35	193^a^	74	0
It is dangerous for people who ejaculate externally to use EOCs	–/–/P/S	240^a^	109	45	17	7	0
It is dangerous for people who use lactating amenorrhea method to use EOCs	–/–/P/S	102	79	77	126^a^	34	0
EOCs do not require medical intervention, e.g., surgery	–/–/–/S	44	67	83	167^a^	56	0–4
EOCs are dangerous	–/–/P/S	25	89	130	142^a^	30	− 4 to 0
EOCs are effective	–/–/P/S	7	30	97	254^a^	25	0–4
EOCs are more effective than combined oral contraceptives	–/–/P/S	76	134^a^	71	115	21	0
EOC use is not complicated	–/–/–/S	13	44	74	245^a^	40	0–4
EOC use is suitable for forgetful people	–/–/P/S	100	124^a^	74	105	14	0–4
EOCs act quickly	–/–/–/S	27	46	126	197^a^	19	0
EOCs ensure contraception	–/–/P/S	55	162^a^	92	98	9	0
EOCs reduce unintentional pregnancy rates	–/I/–/S	10	20	33	267^a^	88	0–4
EOCs reduce the abortion rate	–/I/–/S	19	38	44	238^a^	75	0–4
EOCs encourage rape	–/–/–/S	243^a^	106	29	28	10	− 4 to 0
EOCs are contraception alternatives	–/–/–/S	43	81	57	176^a^	61	0–4
Education about EOCs should be started at the age of …	–/–/P/S	12 years (Q1, Q3 = 12.00, 13.75), *n* = 410					N/A
Pharmacists can refuse EOC dispensing based on their personal beliefs	–/–/–/S	215^a^	141	40	18	2	0
Pharmacists can refuse EOC dispensing based on medical reasons	–/–/–/S	13	11	12	150	232^a^	0
Promoting EOC access also promotes having sex	–/I/–/–	194^a^	158	49	13	4	0–4
EOCs should be sold in universities	–/–/–/S	82	91	70	126^a^	49	0–4
EOCs should be sold in high schools	–/–/–/S	113	141^a^	85	55	22	0–4
All males of reproductive age should be educated on EOCs	–/I/–/S	8	11	26	150	223^a^	0–4
All women of reproductive age should be educated on EOCs	–/I/–/S	8	7	12	123	264^a^	0–4
Sex education should include EOCs	–/I/–/S	10	4	14	138	249^a^	0–4
Pharmacy working hours affect EOC access	–/–/P/S	24	29	83	168^a^	111	0
EOCs should be subsidized and accessed freely	G/–/–/S	37	65	99	113^a^	101	0–4
The price of ECOs is already reasonable	G/I/–/S	8	30	160^a^	154	65	0
How much that the price of EOCs should be (per coitus)?	G/I/–/S	50 Thai Baht (Q1, Q3 = 40, 60), *n* = 396					N/A
EOC users are worried	–/–/P/S	5	19	67	251^a^	76	0
EOC users feel that other people see them negatively	–/I/–/S	22	52	128	172^a^	42	0
EOC users are shy	–/I/P/S	22	41	113	190^a^	49	0
EOCs are in need	–/–/–/S	19	88	130	140^a^	39	0
EOC users should never be judged	–/–/–/S	7	21	51	181^a^	157	0–4
Women should be the sole person who decides if she wants to use EOCs	G/–/–/S	26	113^a^	96	109	73	0
Women should always have EOCs with them	G/–/–/S	37	107	166^a^	77	29	0
There should be a minimum age requirement for EOC use	–/–/P/S	48	121^a^	101	111	34	− 4 to 0
People who seek information on EOCs are going to have sex	–/–/P/–	162^a^	131	70	43	10	− 4 to 0
EOC users should not be refused by pharmacists	–/–/–/S	14	61	60	184^a^	97	0–4
People who do not reach the legal age are perceived negatively by people when they use EOCs	–/–/–/–^b^	54	132^a^	87	127	17	0
People who reach the legal age are perceived negatively by pharmacists when they use EOCs	–/–/–/–	66	80	91	158^a^	21	0
People who do not reach the legal age are perceived negatively by pharmacists when they use EOCs	–/–/–/–	144^a^	125	98	46	5	0
EOC users do not know other contraceptive methods	–/I/–/–	44	145^a^	112	101	15	− 4 to 0
EOC users do not use other contraceptive methods	–/I/–/–	55	174^a^	112	63	12	− 4 to 0
EOC users do not use condoms	–/I/P/S	53	81	80	150^a^	52	− 4 to 0
EOC users change partners frequently	–/I/–/S	84	122	135^a^	54	22	− 4 to 0
EOC users have sex frequently	–/I/–/S	68	129	144^a^	57	17	− 4 to 0
EOC users have sex when they are drunk	–/I/P/S	46	76	136^a^	124	34	− 4 to 0
EOC users are not serious about their relationships	–/I/–/–	63	101	153^a^	87	14	− 4 to 0
EOC users are at risk for sexually transmitted diseases	–/I/–/S	48	50	89	149^a^	82	− 4 to 0
EOCs do not like taking medication every day	–/I/P/S	23	83	114	168^a^	28	0
EOC users take care of themselves	–/I/P/S	43	105	135^a^	105	26	0–4
EOC users are sinful	–/I/P/S	187^a^	118	99	10	1	− 4 to 0
EOC users are responsible	–/I/P/S	16	28	140	168^a^	63	− 4 to 0
EOC users lack knowledge	–/I/P/S	77	122	171^a^	34	12	− 4 to 0
EOC users do not plan	–/I/P/S	43	97	167^a^	84	24	− 4 to 0
You will perceive EOC users negatively when they use EOCs for more than how many doses?	–/–/–/–	2 doses (Q1, Q3 = 0, 2), *n* = 399					N/A
Partners of EOC users should be able to make decisions for EOC users on whether they should use EOCs or not	–/–/–/–	61	111	101	131^a^	13	0
Partners of EOC users affect how EOC users decide to use EOCs	–/I/–/S	44	69	80	191^a^	32	0
Partners of EOC users should know about EOC efficacy	–/I/–/–	0	1	30	203^a^	183	0
Partners of EOC users should know about EOC safety	–/I/–/–	0	2	20	180	214^a^	0
Partners of EOC users who seek EOC knowledge love their partners	–/I/–/–	12	45	166^a^	129	61	0–4
Partners of EOC users who know EOCs are responsible for their partners	–/I/–/–	8	38	107	172^a^	90	0–4
Partners of EOC users who know EOCs are weird	–/–/P/–	178^a^	137	75	18	8	− 4 to 0
If partners of EOC users ask their women to use EOCs, the partners are irresponsible	–/I/–/–	86	112	125^a^	67	26	− 4 to 0
If partners of EOC users ask their women to use EOCs, the partners are selfish	–/–/P/–	81	104	131^a^	71	27	− 4 to 0
Using EOCs is immoral	–/I/–/S	223^a^	122	61	8	2	− 4 to 0
Religious effects on how pharmacists dispense EOCs	–/I/–/S	153^a^	117	86	58	3	0
Religious effects on if people use EOCs	–/I/–/S	110	91	89	111^a^	14	0
Package inserts affect women’s decisions on using EOCs	–/–/–/S	5	26	110	214^a^	63	0
Television media affect women’s decisions on using EOCs	–/–/–/S	14	11	76	239^a^	76	0
Internet and social media affect women’s decisions on using EOCs	–/–/–/S	4	5	34	203^a^	170	0
Partners affect women’s decisions on using EOCs	–/–/–/S	4	11	75	253^a^	74	0
Parents affect women’s decisions on using EOCs	–/–/–/S	5	26	107	212^a^	67	0
Friends affect women’s decisions on using EOCs	–/–/–/S	3	7	58	230^a^	120	0
Physicians affect women’s decisions on using EOCs	–/–/–/S	4	5	59	220^a^	127	0
Pharmacists affect women’s decisions on using EOCs	–/–/–/S	2	4	49	228^a^	133	0
Society affects women’s decisions on using EOCs	–/–/–/S	12	30	203^a^	148	21	0
Selling EOCs should be under the supervision of pharmacists	–/I/–/S	1	0	15	165	236^a^	0
EOCs should be prescribed	–/I/–/S	103	156^a^	105	32	20	0
Dispensing EOCs should get pharmacist fees	–/–/–/S	27	57	207^a^	84	43	0
Pharmacists should counsel EOC buyers	–/–/–/–	2	2	25	128	260^a^	0
Pharmacists should counsel EOC users	–/I/–/–	0	1	19	120	277^a^	0
Dispensing EOCs increases managerial burden	–/–/–/S	37	93	204^a^	75	9	0
Dispensing EOCs takes a long time	–/–/–/S	46	144	162^a^	53	10	0
Buying EOCs from pharmacists of the same sex can reduce shyness	–/–/–/S	12	29	98	208^a^	70	0
Buying EOCs requires privacy	–/–/–/S	3	13	81	239^a^	80	0
Dispensing EOCs is the pharmacists’ responsibility	–/–/–/S	3	5	50	228^a^	129	0
Dispensing EOCs increases pharmacists’ professionalism	–/I/–/S	2	11	171^a^	152	80	0–4
What are the advantages of using telepharmacy for EOC dispensing?	–/I/–/–	Improve EOC access (349/421) Increase convenience to EOC access (349/421) Reduce the cost of transportation/logistics (310/421)					N/A

*G* Google Trend, *I* interviews from the qualitative part, *P* Pantip.com search, *S* the systematic review and meta-analysis, *EOCs* emergency oral contraceptive

^a^Mode

^b^–/–/–/– means that the question is added by either the authors or the experts

**Table 5:** Survey questions for practice during EOC dispensing and their sources, the frequency of the practice, and possible scores

Questions	Source of question	Answer	Possible practice scores
On average, how many doses of EOCs that you dispense per month?	–/–/–/–^b^	4 doses (Q1, Q3 = 0, 15), *n* = 373	N/A
What is the reason that you dispense EOCs?	–/–/–/–	Per prescription: 73/421	N/A
		Per patient request: 335/421^a^	
		Dispense based on patient needs: 99/421	
Have you ever refused to dispense EOCs for the following reason?	–/–/–/–	Never refuse to dispense EOCs: 181/421^a^	N/A
		Use EOCs, not for contraception: 68/421	
		Have contraindications: 155/421	
		Use too frequently: 119/421	
		Risk for sexually transmitted diseases: 16/421	
		Personal reasons: 2/421	
You are going to refuse to dispense EOCs if the patients already use how many doses of ECOs already within this month?	–/–/–/–	4 doses (Q1, Q3 = 2, 4), *n* = 263	N/A

How often do you perform the following practices?	Source of question	Frequency (*n*)					Possible practice scores
		Never	Rarely	Some-times	Mostly	Every-times	
Ask for the reason for using EOCs	–/–/–/–	64	86	57	84	99^a^	0–4
Ask about the history of drug allergies	–/–/–/–	9	21	27	65	269^a^	0–4
Explain how to use EOCs	–/–/–/–	4	6	47	104	231^a^	0–4
Check for information on contraindications	–/–/–/–	10	30	61	107	182^a^	0–4
Refuse to dispense EOCs because of contraindications	–/–/–/–	60	46	35	83	165^a^	0–4
Refuse to dispense EOCs because of personal reasons	–/–/–/–	150^a^	88	40	55	54	− 4–0
Recommend switching to combined oral contraception	–/–/–/–	26	63	91	123^a^	87	0
Recommend concomitant condom use	–/–/–/–	27	45	94	107	116^a^	0–4
Emphasis on taking EOCs on time	–/–/–/–	8	8	42	108	223^a^	0–4
Recommend setting an alarm for the time to take EOCs	–/–/–/–	32	34	73	103	146^a^	0–4
Alert, e.g., sending messages, to patients to take EOCs	–/–/–/–	278^a^	26	37	24	24	0–4

*EOCs* emergency oral contraceptive

^a^Mode

^b^–/–/–/– means that the question is added by either the authors or the experts

### Collecting data for survey construction: Pantip.com searches

We accessed the latest 2000 forums from November 8th, 2020, to April 1st, 2022, and found 82 topics which were then used in 75 survey questions (Tables 3, 4, 5). In addition, text analysis allowed us to extract themes related to EOC knowledge and attitude in Thai internet users. First, sex literacy was low and sex education was inadequate. As a result, the internet was a main source of sexual health information. Second, incorrect information and wrong understanding, e.g., the limited number of EOCs per lifetime, permanent harm from EOCs, coitus interruptus and fertility awareness method as effective contraception, and vaginal bleeding as an indicator for pregnancy, were prevalent.

### Cross-sectional KAP survey

The final cross-sectional survey included 186 questions and was classified into 5 sections. There were 10, 57, 103, and 16 questions for demographics, knowledge, attitudes, and practice, respectively (3, 4 and 5). All of them had an IOC > 0.7. From October 2022 to January 2023, we received 427 responses, but 421 responses were included after data cleaning. The demographics of the participating pharmacists are summarized in Table 6. Briefly, most of the participants were adults; female; graduated with B.Pharm. or Pharm.D.; community or hospital pharmacists; in private section; Buddhists; and single. They were also averagely religious, open to sex discussion, and low or moderately judgmental. More than 90% of them agree that unintentional pregnancy is a social problem (Table 7).

**Table 6:** Demographic data of survey participants

Demographics	Data (*n* = 421)
Median age, years (Q1, Q3)	33, (27, 38)
Sex, *n* (%)	
Female	274 (65.08)
Male	134 (31.83)
Prefer not to answer	13 (3.09)
Degree, *n* (%)	
B.Pharm. or Pharm.D. in clinical pharmacy	244 (57.96)
B.Pharm. or Pharm.D. in pharmaceutical sciences	175 (41.57)
With higher education	158 (37.53)
Occupation, *n* (%)	
Government officer	129 (30.64)
University staff	46 (10.93)
Private section	141 (33.49)
Entrepreneur	88 (20.90)
In education	10 (2.38)
Practice, *n* (%)	
Community pharmacy	145 (34.44)
Hospital pharmacy	143 (33.97)
Other pharmaceutical practice	102 (24.23)
Currently not practicing	30 (7.13)
Median practicing duration, years (Q1, Q3)	1 (4, 10)
Religion, *n* (%)	
No religion	18 (4.27)
Buddhism	386 (91.69)
Christ	11 (2.61)
Islam	4 (0.95)
Others	1 (0.24)
Marital status, *n* (%)	
Single	312
Nonmarriage committed relationship	21
Married	82
Divorce	5

There are 2, 7, 1, 1, and 1 missing data in degree, occupation, practice, religion, and marital status, respectively

**Table 7:** Descriptive statistics for knowledge, attitude, and practice regarding levonorgestrel emergency oral contraceptive

Demographics	Data (*n* = 421)
Awareness, *n* (%)	421 (100.00)
Knowledge	
Possible score (min, max)	(0, 49)
Median knowledge score (q1, q3)	25 (21, 28)
Median knowledge score adjusted as a percentage (q1, q3)	51.02 (42.86, 57.14)
Self-rating knowledge, *n* (%)	
No knowledge	0 (0.00)
Some knowledge	25 (5.94)
Average knowledge	153 (36.34)
Good knowledge	205 (48.69)
Better knowledge	36 (8.55)
Attitude	
Possible score (min, max)	(− 84, 104)
Median attitude score (q1, q3)	41 (29, 55)
Median attitude score adjusted as a percentage (q1, q3)	21.81 (15.42, 29.25)
Self-rating attitude, *n* (%)	
Negative attitude	1 (0.24)
Somewhat negative attitude	27 (6.41)
Neutral attitude	112 (26.60)
Somewhat positive attitude	203 (48.22)
Positive attitude	78 (18.53)
Practice	
Possible score (min, max)	(-4, 36)
Median practice score (q1, q3)	24 (0, 36)
Median practice score adjusted as a percentage (q1, q3)	60.00 (0.00, 100.00)
Self-rating practice, *n* (%)	
Unacceptable practice	0 (0.00)
Somewhat unacceptable practice	3 (0.71)
Acceptable practice	52 (12.35)
Good practice	239 (56.77)
Best practice	126 (29.93)
Religiousness, *n* (%)	
Not at all	49 (11.64)
Barely	35 (8.31)
Somewhat	237 (56.29)
Mostly	88 (20.90)
Absolutely	12 (2.85)
Sex openness, *n* (%)	
Not at all	0 (0.00)
Barely	19 (4.51)
Somewhat	91 (21.62)
Mostly	221 (52.49)
Absolutely	90 (21.38)
Judgmental personality, *n* (%)	
Not at all	72 (17.10)
Barely	156 (37.05)
Somewhat	144 (34.20)
Mostly	45 (10.69)
Absolutely	1 (0.24)
Unintentional pregnancy is a social problem, *n* (%)	
Disagree	5 (1.19)
Somewhat disagree	7 (1.66)
Neutral	16 (3.80)
Somewhat agree	205 (48.69)
Agree	188 (44.66)

There are 2, 1, and 3 missing data in self-rating knowledge, self-rating practice, and judgmental personality, respectively

*q* quartile, *min* minimum, *max* maximum

### Pharmacists’ knowledge of EOCs

All participants were aware of EOCs and 48.69% of them reported good knowledge about EOCs (median knowledge scores = 51.02%; Table 7). We found that, first, the total knowledge score was weakly correlated with the self-rating knowledge score and was not a predictor of the self-rating knowledge score. Second, both total and self-rating knowledge scores were weakly correlated with attitude and practice scores. However, the self-rating knowledge score was moderately associated with the self-rating practice score (ρ = 0.525, *p* < 0.001, Table 8). In addition, the total knowledge score and self-rating knowledge score could explain variances in the total attitude score and self-rating practice score but not the self-rating attitude score and total practice score. Third, graduating with a clinical pharmacy degree, and practicing community or hospital pharmacy was associated with an increased total knowledge score while practicing community pharmacy, not being single, and being open to sex discussion was associated with an increased self-rating knowledge score (Table 8).

**Table 8:** Generalized mixed model for the knowledge, attitude, and practice

Correlation between knowledge, attitude, and practice			
Variables	Self-rating knowledge	Self-rating attitude	Self-rating practice
Self-rating knowledge	ρ = 1.000 (*p* = N/A) *n* = 419	ρ = 0.251 (*p* < 0.001)* *n* = 419	ρ = 0.525 (*p* < 0.001)* *n* = 418
Knowledge	ρ = 0.295 (*p* < 0.001)* *n* = 419	ρ = 0.122 (*p* = 0.012)* *n* = 421	ρ = 0.306 (*p* < 0.001)* *n* = 420
Self-rating attitude	ρ = 0.251 (*p* < 0.001)* *n* = 419	ρ = 1.000 (*p* = N/A)* *n* = 421	ρ = 0.312 (*p* < 0.001)* *n* = 420
Attitude	ρ = 0.175 (*p* < 0.001)* *n* = 419	ρ = 0.307 (*p* < 0.001)* *n* = 421	ρ = 0.107 (*p* = 0.028)* *n* = 420
Self-rating practice	ρ = 0.525 (*p* < 0.001)* *n* = 418	ρ = 0.312 (*p* < 0.001)* *n* = 420	ρ = 1.000 (*p* = N/A)* *n* = 420
Practice	ρ = 0.120 (*p* = 0.018)* *n* = 390	ρ = 0.202 (*p* < 0.001)* *n* = 392	ρ = 0.148 (*p* = 0.003)* *n* = 392

Multivariable analysis of the total score of knowledge, attitude, and practice^a^			
Variables	Total knowledge score	Total attitude score	Total practice score
Sex	*B* = 0.947 ± 0.536 (*p* = 0.078)	*B* =− 1.747 ± 1.818 (*p* = 0.337)	*B* = 0.617 ± 0.700 (*p* = 0.378)
Age	*B* =− 0.019 ± 0.061 (*p* = 0.751)	*B* =− 0.328 ± 0.205 (*p* = 0.109)	*B* = − 0.111 ± 0.079 (*p* = 0.160)
Degree	*B* = 1.770 ± 0.573 (*p* = 0.002)*	*B* =− 2.655 ± 1.958 (*p* = 0.175)	*B* =− 0.869 ± 0.754 (*p* = 0.249)
Setting	*B*_1_ = 3.676 ± 0.729 (*p* < 0.001)* *B*_2_ = 2.415 ± 0.762 (*p* = 0.002)*	*B*_1_ =− 8.080 ± 2.510 (*p* = 0.001)* *B*_2_ =− 6.968 ± 2.586 (*p* = 0.007)*	*B*_1_ =− 2.734 ± 0.969 (*p* < 0.005)* *B*_2_ = 0.188 ± 1.005 (*p* = 0.852)
Practice duration	*B* = 0.075 ± 0.070 (*p* = 0.281)	*B* =− 0.014 ± 0.236 (*p* = 0.952)	*B* = 0.008 ± 0.091 (*p* = 0.929)
Religion	*B* = 0.384 ± 0.922 (*p* = 0.677)	*B* = 0.907 ± 3.115 (*p* = 0.771)	*B* =− 0.331 ± 1.199 (*p* = 0.796)
Religiousness	*B* = 0.164 ± 0.283 (*p* = 0.563)	*B* =− 3.145 ± 0.941 (*p* = 0.001)*	*B* =− 0.210 ± 0.368 (*p* = 0.567)
Marital status	*B* =− 0.511 ± 0.649 (*p* = 0.431)	*B* = 4.538 ± 2.181 (*p* = 0.037)*	*B* = 0.526 ± 0.844 (*p* = 0.533)
Openness	*B* = 0.690 ± 0.362 (*p* = 0.057)	*B* = 2.020 ± 1.225 (*p* = 0.099)	*B* = 1.212 ± 0.469 (*p* = 0.010)*
Judgmental	*B* =− 0.150 ± 0.284 (*p* = 0.599)	*B* =− 0.401 ± 0.961 (*p* = 0.677)	*B* = 0.037 ± 0.370 (*p* = 0.920)
Unwanted pregnancy	*B* = 0.207 ± 0.361 (*p* = 0.566)	*B* = 1.450 ± 1.218 (*p* = 0.234)	*B* =− 1.091 ± 0.466 (*p* = 0.019)*
Self-rating knowledge	*B* = 0.168 ± 0.428 (*p* = 0.695)	*B* = 2.962 ± 1.439 (*p* = 0.040)*	*B* = 0.509 ± 0.556 (*p* = 0.360)
Knowledge	N/A	*B* = 0.703 ± 0.176 (*p* < 0.001)*	*B* = 0.097 ± 0.069 (*p* = 0.159)
Self-rating attitude	*B* = − 0.504 ± 0.344 (*p* = 0.142)	*B* = 5.701 ± 1.124 (*p* < 0.001)*	*B* = 1.108 ± 0.444 (*p* = 0.013)*
Attitude	*B* = 0.062 ± 0.015 (*p* < 0.001)*	N/A	*B* = 0.002 ± 0.020 (*p* = 0.909)
Self-rating practice	*B* = 1.444 ± 0.499 (*p* = 0.004)*	*B* =− 2.367 ± 1.700 (*p* = 0.164)	*B* = 0.747 ± 0.655 (*p* = 0.254)
Practice	*B* = 0.057 ± 0.041 (*p* = 0.159)	*B* = 0.016 ± 0.138 (*p* = 0.909)	N/A

Multivariable analysis of the self-rating of knowledge, attitude, and practice score^b^			
Variables	Self-rating knowledge score	Self-rating attitude score	Self-rating practice score
Sex	*B* =− 0.056 ± 0.067 (*p* = 0.400)	*B* =− 0.051 ± 0.083 (*p* = 0.538)	*B* = 0.076 ± 0.057 (*p* = 0.179)
Age	*B* =− 0.004 ± 0.007 (*p* = 0.554)	*B* =− 0.005 ± 0.009 (*p* = 0.631)	*B* = − 0.016 ± 0.006 (*p* = 0.014)*
Degree	*B* = 0.059 ± 0.072 (*p* = 0.415)	*B* = 0.097 ± 0.089 (*p* = 0.277)	*B* = − 0.040 ± 0.061 (*p* = 0.510)
Setting	*B*_1_ = 0.270 ± 0.092 (*p* = 0.004)* *B*_2_ = 0.007 ± 0.096 (*p* = 0.941)	*B*_1_ = 0.094 ± 0.116 (*p* = 0.418) *B*_2_ = 0.073 ± 0.119 (*p* = 0.543)	*B*_1_ = − 0.003 ± 0.079 (*p* = 0.967) *B*_2_ = 0.043 ± 0.081 (*p* = 0.597)
Practice duration	*B* = 0.007 ± 0.008 (*p* = 0.430)	*B* = 0.006 ± 0.011 (*p* = 0.593)	*B* = 0.006 ± 0.007 (*p* = 0.427)
Religion	*B* =− 0.181 ± 0.114 (*p* = 0.112)	*B* =− 0.325 ± 0.141 (*p* = 0.021)*	*B* = 0.219 ± 0.097 (*p* = 0.023)*
Religiousness	*B* =− 0.031 ± 0.035 (*p* = 0.383)	*B* = 0.072 ± 0.043 (*p* = 0.098)	*B* = 0.105 ± 0.029 (*p* < 0.001)*
Marital status	*B* =− 0.186 ± 0.080 (*p* < 0.001)*	*B* = 0.191 ± 0.100 (*p* = 0.055)	*B* = − 0.124 ± 0.068 (*p* = 0.069)
Openness	*B* = 0.053 ± 0.045 (*p* = 0.020)*	*B* = 0.090 ± 0.056 (*p* = 0.109)	*B* = − 0.020 ± 0.038 (*p* = 0.611)
Judgmental	*B* = − 0.042 ± 0.035 (*p* = 0.228)	*B* = − 0.022 ± 0.044 (*p* = 0.621)	*B* = − 0.039 ± 0.030 (*p* = 0.197)
Unwanted pregnancy	*B* =− 0.061 ± 0.045 (*p* = 0.175)	*B* = 0.065 ± 0.056 (*p* = 0.241)	*B* = 0.120 ± 0.038 (*p* = 0.001)*
Self-rating knowledge	N/A	*B* = 0.067 ± 0.066 (*p* = 0.309)	*B* = 0.369 ± 0.041 (*p* < 0.001)*
Knowledge	*B* = 0.003 ± 0.007 (*p* = 0.695)	*B* = − 0.012 ± 0.008 (*p* = 0.142)	*B* = 0.016 ± 0.005 (*p* = 0.004)*
Self-rating attitude	*B* = 0.044 ± 0.043 (*p* = 0.309)	N/A	*B* = 0.151 ± 0.035 (*p* < 0.001)*
Attitude	*B* = 0.004 ± 0.002 (*p* = 0.040)*	*B* = 0.012 ± 0.003 (*p* < 0.001)*	*B* = − 0.002 ± 0.002 (*p* = 0.164)
Self-rating practice	*B* = 0.513 ± 0.056 (*p* < 0.001)*	*B* = 0.324 ± 0.076 (*p* < 0.001)*	N/A
Practice	*B* = 0.005 ± 0.005 (*p* = 0.360)	*B* = 0.016 ± 0.006 (*p* = 0.013)*	*B* = 0.005 ± 0.004 (*p* = 0.254)

^a^The dependent variables were total scores of knowledge or attitude or practice. Independent variables were sex (female, male), age (years), degree (clinical pharmacy, others), setting (community_1_, hospital_2_, others), practice duration (years), religion (Buddhist, others), marital status (single, others), self-rating religiousness, self-rating openness to sex discussion, self-rating judgmental personality, self-rating attitude toward unwanted pregnancy, self-rating scores of knowledge, attitude, and practice

^b^The dependent variables were self-rating scores of knowledge or attitude or practice. Independent variables were sex (female, male), age (years), degree (clinical pharmacy, others), setting (community_1_, hospital_2_, others), practice duration (years), religion (Buddhist, others), marital status (single, others), self-rating religiousness, self-rating openness to sex discussion, self-rating judgmental personality, self-rating attitude toward unwanted pregnancy, total scores of knowledge, attitude, and practice

ρ: Spearman’s correlation coefficient; *p < 0.05

In this study, less than 50% of the pharmacists knew the correct answer for the tradename, maximum doses for continuous use, the window period for repeating doses after vomiting, efficacy reduction after frequent use, the effective period after a complete dose, teratogenicity, relationship with abortifacient action, and drug interaction. In addition, less than 25% knew the correct answer for the mechanism of action, maximum doses per month, effectiveness after ovulation, the window period for side effects after a complete dose, serious side effects that required EOC cessation, and details in menstrual disturbances (Table 3). Moreover, the knowledge of EOC contraindications should be questioned since 62.94% and 64.13% of the participants knew that breast cancer and severe hepatic impairment, respectively, was a contraindication for levonorgestrel, while 66.50%, 46.32%, and 25.65% of the participants misunderstood that thromboembolism, ectopic pregnancy, and hypertension, respectively, was the contraindication (Table 3).

### Pharmacists’ attitude toward EOCs

Most of the participants (48.22%) reported a somewhat positive attitude toward EOCs (median attitude scores = 21.81%; Table 7). We found that, first, the total attitude score and self-rating attitude were weakly correlated (ρ = 0.307, *p* < 0.001) (Table 8). However, the total attitude score significantly explained the self-rating attitude score (*p* < 0.001), and vice versa (*p* < 0.001). Second, the total attitude score did not explain variances between the total practice score (*p* = 0.909) and self-rating practice score (*p* = 0.164), but the self-rating attitude score explained the total practice score (*p* = 0.013) and the self-rating practice score (*p* < 0.001; Table 8). Finally, practicing community or hospital pharmacy, being religious, and not being single were associated with a lower total attitude score, while being non-Buddhist was associated with a lower self-rating attitude score (Table 8).

Attitudes expressed by the participants were summarized (Table 4). Pharmacists agreed that (1) EOCs should not be used with the lactational amenorrhea method and combined oral contraceptives because of safety concerns; (2) EOCs should be used when ejaculation occurred; (3) EOCs were a fast-acting, effective, and dangerous contraception alternative; and (4) EOC knowledge should be promoted in anyone at puberty ages, confirming the themes found during the in-depth interview. Although pharmacists did not agree with EOC use restrictions based on age, they felt that selling EOCs in schools was not appropriate, while selling EOCs in universities was acceptable.

Data from both the in-depth interview and survey showed that, to pharmacists, using EOCs showed responsibility. They perceived that EOC users were shy and had negative feelings toward EOC use, which could be a barrier to EOC use. Most pharmacists did not feel that they were the barrier to EOC use or that EOCs should be prescription drugs. Pharmacists felt that dispensing EOCs was their responsibility, and selling EOCs should be under their supervision. Most of them did not approve of EOC refusal because of personal reasons. However, EOC refusal based on medical reasons was acceptable. This finding agreed with the qualitative study.

From pharmacists' viewpoints, package inserts, media, partners, parents, friends, and healthcare professionals affected the decision-making of EOC users. Pharmacists felt that responsible male partners would have active roles in obtaining knowledge and making the decision on EOC use. Eighty-five percent (356/417) of the pharmacists reported that religion did not affect their decision on EOC dispensing. Less than 3% of Thai pharmacists perceived using EOCs as immoral or sinful. However, some of them felt that religion did affect whether patients used EOCs or not. Although pharmacists' attitudes toward EOC use and promiscuity or other sex behaviors were neutral, they perceived that EOC use increased the risk of sexually transmitted diseases.

### Pharmacists’ practice on EOCs

Most of the participants (56.77%) reported that they had good pharmacy practice during EOC dispensing (median practice scores = 60%; Table 7). We found that, first, the correlation between the total practice score and self-rating practice score was significant but very weak (ρ = 0.148, *p* = 0.003). The total practice score did not explain the variances of the self-rating practice score and vice versa. Second, practicing community pharmacy was associated with decreased total practice score, while being more open to sex discussion and not seeing unwanted pregnancy as problems were associated with an increased total practice score. In addition, being younger, being non-Buddhist, defining oneself as religious, and viewing unwanted pregnancy as problems were associated with an increased self-rating practice score (Table 8).

More than 80% of pharmacists reported that they dispensed EOCs, because their patients requested the EOCs. Approximately 40% of them reported that they never refuse to dispense EOCs. The major reason for EOC refusal is contraindications. In addition, 60% of them reported they would not have dispensed EOCs for the patients had the patients already used 3 mg levonorgestrel EOCs within that month. Pharmacists had very diverse practices of asking for the reason for EOC requests, ranging from never asking to always asking. Half of the participants would recommend switching EOCs to combine oral contraceptives and recommend concomitant condom use. Pharmacists frequently emphasized to their patients to take EOCs on time or suggested their patients set an alarm. Sending reminder messages was rarely practiced (Table 5).

## Discussion

In Thailand, levonorgestrel EOCs can be sold by pharmacists without prescriptions since 1997 (the Order of Ministry of Public Health 1037/2543). Contraception is also listed as one of the core competencies for Thai pharmacists since 2002 [15]. The long experiences with EOCs and compulsory education on contraception may lead to the 100% EOC awareness found in this study, which was higher than what was reported in a meta-analysis [8]. However, this did not change the fact that EOCs are still misunderstood and misperceived by pharmacists, as supported by findings from the interview and the survey. The knowledge scores were not as high as reported in the previous study [8], as we expected, since we asked pharmacists highly detailed oriented real-world questions from EOC users. In addition, most of the participants reported a neutral or positive attitude toward EOCs. In this study, refusing to dispense EOCs because of nonmedical reasons was perceived as “unacceptable”. However, pharmacists imprecisely identified thromboembolism, ectopic pregnancy, and hypertension as levonorgestrel contraindications. Misunderstanding the contraindications of EOC can potentially pose a barrier to EOC access.

We found that the self-rating KAP scores were higher than the total KAP scores. In addition, using total scores or self-rating scores led to different conclusions on the relationship between KAP. For example, the total practice score could not be predicted by either the total knowledge score or the total attitude score, but the self-rating practice score could be predicted by self-rating knowledge and self-rating attitude. Several other KAP surveys used a summation of correct answers among knowledge, attitude, and practices [16–18], and a guideline to conduct a KAP survey also recommends using the total score [9]. We used both approaches and found that how pharmacists perceive their knowledge and attitude toward EOCs affected how they perceive their practice, but the knowledge and attitude did not affect their practice.

Several variables affect the total scores of KAP. First, pharmacists who practiced in pharmacies and hospitals had a higher total knowledge score than those who practiced in other settings (e.g., manufacturing plants or regulatory offices). A degree in clinical pharmacy is also associated with higher knowledge scores, agreeing to a study in Nepal [19]. Second, even though religion may have affected self-rating attitude or practice scores, it did not affect total KAP scores. This agrees with a study on Christian pharmacy students which also found that religion did not affect the attitude toward EOCs [20]. Third, the year of practice, which was found to be associated with dispensing practice [21], did not explain the practice in our study. This might be because all registered pharmacists in Thailand must participate in annual Continuing Pharmacy Education (CPE).

The major limitation of this study was that the survey was very long. Other limitations due to the cross-sectional nature of this survey were that, first, this survey might not accurately reflect temporal variation in pharmacists' KAP. Second, recall bias may occur with some questions that require recollection of the data retrospectively. Third, using online surveys can lead to the selection of younger pharmacists who are more compatible with using the internet. Despite these, our study has several implications. We have several positive feedbacks suggesting that distributing the survey answers (Table 3) can help correct EOC misunderstandings and can be helpful in pharmacy education and training. For other applications, we provide preliminary evidence that the relationship between KAP yielded from the total scores and self-rating scores can disagree. In addition, even when the EOC can be accessed without prescription, the awareness reaches 100%, and the self-reported attitude toward EOCs among pharmacists is positive, myths that prevent EOC access, especially those regarding contraindications, can be prevalent. This highlights the importance of providing updated knowledge relevant to EOC among Thai pharmacists through a variety of channels including CPE. For further application of this study, since education and economics affected EOC awareness [8], this study should represent Buddhist countries with middle levels of income and education, where EOCs can be accessed without prescriptions.

## Conclusions

Most of the access to EOCs in Thailand is derived from patient requests to pharmacists. Therefore, Thai pharmacists play important roles in the care and promotion of EOC use. We found discrepancies between self-rating KAP scores and total KAP scores. Pharmacists rated their KAP scores higher than their actual KAP scores. The correlation among KAP components was weak. Self-rating scores showed that knowledge and attitudes were associated with the practice, but the total scores showed that the knowledge of or attitude toward EOCs was not associated with EOC practice. EOC knowledge and attitudes should be promoted to prevent refusal of EOC dispensing but this may not improve EOC practice.

## Supplementary Information

**Additional file 1**: A list of interview questions for thesemi-structured interview in the qualitative research.

**Additional file 2**: References for the answer to the knowledge survey.

## Data Availability

The data sets used and/or analyzed during the current study are available from the corresponding author upon reasonable request.

## Abbreviations

B.Pharm: Bachelor of Pharmacy
CPE: Continuing pharmacy education
EOC: Emergency oral contraception
IOC: Index of item objective congruence
KAP: Knowledge, attitude, and practice
Pharm.D: Doctor of Pharmacy
RA: Research assistant
SPV: Subjects per variable
