# Supplementary material for: After morning, phew! A knowledge, attitudes, and practices survey related to emergency oral contraception in Thai pharmacists
Source: J Pharm Policy Pract. 2023 Aug 1;16:97. doi: 10.1186/s40545-023-00601-9 (PMC10391845; doi:10.1186/s40545-023-00601-9)
Supplement: Supplementary file 1 — Additional file 1: A list of interview questions for thesemi-structured interview in the qualitative research. [file 40545_2023_601_MOESM1_ESM.docx]

Additional file 1: Data 1. A list of interview questions for the semi-structured interview in the qualitative research

1. What do you think pharmacists should know about emergency oral contraceptives (EOCs)? Please tell us as complete as possible.

2. What do you think EOC users should know about emergency oral contraceptives? Please tell us as complete as possible.

3. How do you feel about using EOCs.?

4. What makes people use EOCs?

5. What makes people not use EOCs?

6. What are the consequences of using EOCs?
