# Supplementary material for: After morning, phew! A knowledge, attitudes, and practices survey related to emergency oral contraception in Thai pharmacists
Source: J Pharm Policy Pract. 2023 Aug 1;16:97. doi: 10.1186/s40545-023-00601-9 (PMC10391845; doi:10.1186/s40545-023-00601-9)
Supplement: Supplementary file 2 — Additional file 2: References for the answer to the knowledge survey. [file 40545_2023_601_MOESM2_ESM.docx]

Additional file 2: Data 2. References for the answer to the knowledge survey

Altshuler, A. L., Gaffield, M. E., & Kiarie, J. N. (2015). The WHO's medical eligibility criteria for contraceptive use: 20 years of global guidance. *Curr Opin Obstet Gynecol*, *27*(6), 451-459. <https://doi.org/10.1097/gco.0000000000000212>

American Pharmacist Association. (2018). *Drug Information Handbook with International Trade Names Index* (27th ed.). Wolters Kluwer.

Endler, M., Li, R. H. W., & Gemzell Danielsson, K. (2022). Effect of levonorgestrel emergency contraception on implantation and fertility: A review. *Contraception*, *109*, 8-18. <https://doi.org/10.1016/j.contraception.2022.01.006>

Gainer, E., Kenfack, B., Mboudou, E., Doh, A. S., & Bouyer, J. (2006). Menstrual bleeding patterns following levonorgestrel emergency contraception. *Contraception*, *74*(2), 118-124. <https://doi.org/10.1016/j.contraception.2006.02.009>

Gemzell-Danielsson, K., Berger, C., & P.G.L, L. (2013). Emergency contraception - mechanisms of action. *Contraception*, *87*(3), 300-308. <https://doi.org/10.1016/j.contraception.2012.08.021>

Gemzell-Danielsson, K., Kardos, L., & von Hertzen, H. (2015). Impact of bodyweight/body mass index on the effectiveness of emergency contraception with levonorgestrel: a pooled-analysis of three randomized controlled trials. *Current Medical Research and Opinion*, *31*(12), 2241-2248. <https://doi.org/10.1185/03007995.2015.1094455>

Graña, C., Ghosn, L., Evrenoglou, T., Jarde, A., Minozzi, S., Bergman, H., Buckley, B. S., Probyn, K., Villanueva, G., Henschke, N., Bonnet, H., Assi, R., Menon, S., Marti, M., Devane, D., Mallon, P., Lelievre, J. D., Askie, L. M., Kredo, T., Ferrand, G., Davidson, M., Riveros, C., Tovey, D., Meerpohl, J. J., Grasselli, G., Rada, G., Hróbjartsson, A., Ravaud, P., Chaimani, A., & Boutron, I. (2022). Efficacy and safety of COVID-19 vaccines. *Cochrane Database Syst Rev*, *12*(12), Cd015477. <https://doi.org/10.1002/14651858.Cd015477>

Halpern, V., Raymond, E. G., & Lopez, L. M. (2014). Repeated use of pre- and postcoital hormonal contraception for prevention of pregnancy. *Cochrane Database Syst Rev*, *2014*(9), Cd007595. <https://doi.org/10.1002/14651858.CD007595.pub3>

Hapangama, D., Glasier, A. F., & Baird, D. T. (2001). The effects of peri-ovulatory administration of levonorgestrel on the menstrual cycle. *Contraception*, *63*(3), 123-129. <https://doi.org/10.1016/s0010-7824(01)00186-x>

Johansson, E., Brache, V., Alvarez, F., Faundes, A., Cochon, L., Ranta, S., Lovern, M., & Kumar, N. (2002). Pharmacokinetic study of different dosing regimens of levonorgestrel for emergency contraception in healthy women. *Hum Reprod*, *17*(6), 1472-1476. <https://doi.org/10.1093/humrep/17.6.1472>

Kardos, L. (2020). Levonorgestrel emergency contraception and bodyweight: are current recommendations consistent with historic data? *Journal of Drug Assessment*, *9*(1), 37-42. <https://doi.org/10.1080/21556660.2020.1725524>

Kives, S., Hahn, P. M., White, E., Stanczyk, F. Z., & Reid, R. L. (2005). Bioavailability of the Yuzpe and levonorgestrel regimens of emergency contraception: vaginal vs. oral administration. *Contraception*, *71*(3), 197-201. <https://doi.org/10.1016/j.contraception.2004.09.009>

Kook, K., Gabelnick, H., & Duncan, G. (2002). Pharmacokinetics of levonorgestrel 0.75 mg tablets. *Contraception*, *66*(1), 73-76. <https://doi.org/10.1016/s0010-7824(02)00321-9>

Kovacs, G. T., Hendricks, J., Summerbell, D., & Baker, H. W. (2000). A pre-coital pill? A preliminary in vitro study. *Br J Fam Plann*, *26*(3), 165-166. <https://doi.org/10.1783/147118900101194418>

Lee, J. H., Song, J. Y., Yi, K. W., Kim, J. J., Hwang, K. R., Shin, J. H., Lee, J. Y., & Chae, H. D. (2022). Contraception in the COVID-19 pandemic: recommendations from the Korean society of contraception and reproductive health. *Obstet Gynecol Sci*, *65*(2), 125-132. <https://doi.org/10.5468/ogs.21322>

Leelakanok, N., & Methaneethorn, J. (2020). A Systematic Review and Meta-analysis of the Adverse Effects of Levonorgestrel Emergency Oral Contraceptive. *Clin Drug Investig*, *40*(5), 395-420. <https://doi.org/10.1007/s40261-020-00901-x>

Matyanga, C. M. J., & Dzingirai, B. (2018). Clinical Pharmacology of Hormonal Emergency Contraceptive Pills. *Int J Reprod Med*, *2018*, 2785839. <https://doi.org/10.1155/2018/2785839>

Noé, G., Croxatto, H. B., Salvatierra, A. M., Reyes, V., Villarroel, C., Muñoz, C., Morales, G., & Retamales, A. (2011). Contraceptive efficacy of emergency contraception with levonorgestrel given before or after ovulation. *Contraception*, *84*(5), 486-492. <https://doi.org/10.1016/j.contraception.2011.03.006>

Okewole, I. A., Arowojolu, A. O., Odusoga, O. L., Oloyede, O. A., Adeleye, O. A., Salu, J., & Dada, O. A. (2007). Effect of single administration of levonorgestrel on the menstrual cycle. *Contraception*, *75*(5), 372-377. <https://doi.org/10.1016/j.contraception.2007.01.019>

Raymond, E. G., Halpern, V., & Lopez, L. M. (2011). Pericoital oral contraception with levonorgestrel: a systematic review. *Obstet Gynecol*, *117*(3), 673-681. <https://doi.org/10.1097/AOG.0b013e318209dc25>

Rott, H. (2019). Birth Control Pills and Thrombotic Risks: Differences of Contraception Methods with and without Estrogen. *Hamostaseologie*, *39*(1), 42-48. <https://doi.org/10.1055/s-0039-1677806>

Sunaga, T., Cicali, B., Schmidt, S., & Brown, J. (2021). Comparison of contraceptive failures associated with CYP3A4-inducing drug-drug interactions by route of hormonal contraceptive in an adverse event reporting system. *Contraception*, *103*(4), 222-224. <https://doi.org/https://doi.org/10.1016/j.contraception.2020.12.002>

Taylor, D. J., Lendvay, A., Halpern, V., Bahamondes, L. G., Fine, P. M., Ginde, S. Y., Wheeless, A., & Raymond, E. G. (2014). A single-arm study to evaluate the efficacy, safety and acceptability of pericoital oral contraception with levonorgestrel. *Contraception*, *89*(3), 215-221. <https://doi.org/10.1016/j.contraception.2013.11.013>

Tremblay, D., Gainer, E., & Ulmann, A. (2001). The pharmacokinetics of 750 microg levonorgestrel following administration of one single dose or two doses at 12- or 24-h interval. *Contraception*, *64*(6), 327-331. <https://doi.org/10.1016/s0010-7824(01)00276-1>

Trussell, J. (2009). Understanding contraceptive failure. *Best Pract Res Clin Obstet Gynaecol*, *23*(2), 199-209. <https://doi.org/10.1016/j.bpobgyn.2008.11.008>

Trussell, J., & Portman, D. (2013). The creeping Pearl: Why has the rate of contraceptive failure increased in clinical trials of combined hormonal contraceptive pills? *Contraception*, *88*(5), 604-610. <https://doi.org/10.1016/j.contraception.2013.04.001>

United Nations Development Programme, United Nations Population Fund, World Health Organization, World Bank Special Programme of Research, Development and Research Training in Human Reproduction, & Task Force on Post-Ovulatory Methods of Fertility Regulation. (2000). Efficacy and side effects of immediate postcoital levonorgestrel used repeatedly for contraception. . *Contraception*, *61*(5), 303-308.

Vargas, M. F., Tapia-Pizarro, A. A., Henríquez, S. P., Quezada, M., Salvatierra, A. M., Noe, G., Munroe, D. J., Velasquez, L. A., & Croxatto, H. B. (2012). Effect of single post-ovulatory administration of levonorgestrel on gene expression profile during the receptive period of the human endometrium. *J Mol Endocrinol*, *48*(1), 25-36. <https://doi.org/10.1530/jme-11-0094>

von Hertzen, H., Piaggio, G., Peregoudov, A., Ding, J., Chen, J., Song, S., Bártfai, G., Ng, E., Gemzell-Danielsson, K., Oyunbileg, A., Wu, S., Cheng, W., Lüdicke, F., Pretnar-Darovec, A., Kirkman, R., Mittal, S., Khomassuridze, A., & Apter, D. (2002). Low dose mifepristone and two regimens of levonorgestrel for emergency contraception: a WHO multicentre randomised trial. *The Lancet*, *360*(9348), 1803-1810. <https://doi.org/10.1016/S0140-6736(02)11767-3>

Vrettakos, C., & Bajaj, T. (2022). Levonorgestrel (*StatPearls*. StatPearls Publishing.

Wai Ngai, S., Fan, S., Li, S., Cheng, L., Ding, J., Jing, X., Yu Ng, E. H., & Chung Ho, P. (2005). A randomized trial to compare 24 h versus 12 h double dose regimen of levonorgestrel for emergency contraception. *Human Reproduction*, *20*(1), 307-311. <https://doi.org/10.1093/humrep/deh583>

World Health, O. (2019). Hepatitis B vaccines: WHO position paper, July 2017 - Recommendations. *Vaccine*, *37*(2), 223-225. <https://doi.org/10.1016/j.vaccine.2017.07.046>

World Health Organization. (2017). Human papillomavirus vaccines: WHO position paper, May 2017-Recommendations. *Vaccine*, *35*(43), 5753-5755. <https://doi.org/10.1016/j.vaccine.2017.05.069>

Zhao, R., Wu, J.-Q., Li, Y.-Y., Zhou, Y., Ji, H.-L., & Li, Y.-R. (2014). Efficacy of a combined contraceptive regimen consisting of condoms and emergency contraception pills. *BMC Public Health*, *14*(1), 354. <https://doi.org/10.1186/1471-2458-14-354>
